# Leveraging Generative AI Tools to Support the Development of Digital Solutions in Health Care Research: Case Study

**DOI:** 10.2196/52885

**Published:** 2024-03-06

**Authors:** Danissa V Rodriguez, Katharine Lawrence, Javier Gonzalez, Beatrix Brandfield-Harvey, Lynn Xu, Sumaiya Tasneem, Defne L Levine, Devin Mann

**Affiliations:** 1 Department of Population Health New York University Grossman School of Medicine New York, NY United States; 2 Medical Center Information Technology Department of Health Informatics New York University Langone Health New York, NY United States

**Keywords:** digital health, GenAI, generative, artificial intelligence, ChatGPT, software engineering, mHealth, mobile health, app, apps, application, applications, diabetes, diabetic, diabetes prevention, digital prescription, software, engagement, behaviour change, behavior change, developer, developers, LLM, LLMs, language model, language models, NLP, natural language processing

## Abstract

**Background:**

Generative artificial intelligence has the potential to revolutionize health technology product development by improving coding quality, efficiency, documentation, quality assessment and review, and troubleshooting.

**Objective:**

This paper explores the application of a commercially available generative artificial intelligence tool (ChatGPT) to the development of a digital health behavior change intervention designed to support patient engagement in a commercial digital diabetes prevention program.

**Methods:**

We examined the capacity, advantages, and limitations of ChatGPT to support digital product idea conceptualization, intervention content development, and the software engineering process, including software requirement generation, software design, and code production. In total, 11 evaluators, each with at least 10 years of experience in fields of study ranging from medicine and implementation science to computer science, participated in the output review process (ChatGPT vs human-generated output). All had familiarity or prior exposure to the original personalized automatic messaging system intervention. The evaluators rated the ChatGPT-produced outputs in terms of understandability, usability, novelty, relevance, completeness, and efficiency.

**Results:**

Most metrics received positive scores. We identified that ChatGPT can (1) support developers to achieve high-quality products faster and (2) facilitate nontechnical communication and system understanding between technical and nontechnical team members around the development goal of rapid and easy-to-build computational solutions for medical technologies.

**Conclusions:**

ChatGPT can serve as a usable facilitator for researchers engaging in the software development life cycle, from product conceptualization to feature identification and user story development to code generation.

**Trial Registration:**

ClinicalTrials.gov NCT04049500; https://clinicaltrials.gov/ct2/show/NCT04049500

## Introduction

Health care has undergone a digital transformation, resulting in a growing reliance on software engineering for medical use cases, including health care research. However, little guidance exists for health researchers on how to effectively develop digital health interventions [1]; in particular, software development challenges that include expertise gaps in coding, custom development needs, high costs, and time constraints result in multilevel barriers to designing and deploying a usable, scalable, and sustainable digital health product [1].

Generative artificial intelligence (GenAI) technologies such as ChatGPT can potentially support researchers in health technology endeavors by providing foundational frameworks and processes for the software development life cycle [2]. These systems can help reduce time and enhance precision for technology-based research projects by supporting both nonprogrammers and experienced programmers in code development, troubleshooting, and cleaning [2]. Moreover, the ability to use GenAI to generate content from different perspectives (expert or nonexpert) can facilitate and improve communication between technical and nontechnical team members of multidisciplinary teams. For example, a nontechnical team member can write their ideas in natural text and then use GenAI to request assistance in creating discussion points to communicate to a technical team audience. GenAI tools may also help health technology researchers refine research questions, identify appropriate theoretical frameworks and models, and leverage popular implementation strategies such as design thinking to build effective, theory-grounded, and evidence-based digital health interventions. ChatGPT (OpenAI, Microsoft Corporation) has already demonstrated feasibility as a support tool for clinical decision support development in health care [3], and more broadly as a coding copilot in programming and engineering [4,5].

This study explores the use of ChatGPT to recreate a personalized automatic messaging system (PAMS), which was developed as part of a digital health research initiative to support patient engagement with a commercial digital diabetes prevention program (dDPP). We examine the capacity, advantages, and limitations of ChatGPT to support product ideation and conceptualization, intervention content development, and the software engineering process including software requirement generation, software design, and code production. This paper provides insights to support the GenAI-assisted development of computational tools that are usable, reliable, extensible, and in line with the standards of modern coding practices. The framework includes prompts for both the intervention conceptualization as well as the main phases of the software development process.

## Methods

### Settings and Intervention Development Context

In previous work [6], we described the development of PAMS, a novel integrated multicomponent communications platform, to promote patient-provider communication and patient engagement in a commercial dDPP (Noom; Noom, Inc). The PAMS intervention included early prototyping and user testing, a technical development phase, and a randomized controlled trial. The core content and user experience features of PAMS were identified, prototyped, and evaluated using the well-established design thinking “discover, define, design, and test” approach to iteratively gather information, define, design, and refine the engagement intervention [7]. Stakeholders included: patients with prediabetes and their support network (eg, caregivers and partners), primary care providers, health technologists, programmers and computer scientists, behavioral change theorists and subject matter experts, the research administrative team, and dDPP product developers and coaches. The main components of this PAMS intervention include (1) a theory-driven behavior change messaging library, (2) a personalized automated message system delivery platform (SMS text messaging–based), and (3) EHR-integrated data visualizations. The PAMS messaging library uses an integrated framework that combines established theoretical models for behavior change with human-centered design strategies to maximize the evidence-based conditions for behavior change and the user acceptance and use of a digital health product. The technical development of PAMS followed an agile software development approach based on incremental 2-week sprint cycles consisting of requirement planning, design, development, and testing of a specific set of functional features. In this paper, we will recreate this development process using GenAI (ChatGPT).

### ChatGPT-PAMS Experiment Design

To evaluate the effectiveness of using GenAI to support the development of digital tools in medical settings, our experiment is based on recreating PAMS using GenAI (ChatGPT) and evaluating human-generated vs ChatGPT-generated documentation. To accurately capture the ideation and development process, our multidisciplinary team reviewed all documentation and processes used in the early stages of PAMS conceptualization, including supporting theoretical models, content and features, and technical development. We then recreated these processes via a series of prompts for ChatGPT-4 to assist with the generation of theory, content, user stories, requirement documents, design diagrams, and the code for a subset of the requirements. Outputs from ChatGPT were reviewed and compared to human-generated documentation by 11 evaluating team members. Evaluators consisted of clinicians, behavioral scientists, programmers, and research staff working in digital health and technology for behavior change research. Collectively, they represent more than 50 years of clinical, research, design, and computer science experience. The evaluators independently rated the quality of various aspects of information provided by ChatGPT on a Likert scale, where higher ratings indicated greater quality of information (*1: very poor; 2: poor; 3: acceptable; 4: good; 5: very good; N/A: not applicable*). Aspects of evaluation included: understandability (Does this output make sense given the context of the study and prompts?), novelty (Were new ideas generated?) [3], usability (Does this create a usable output?), relevance (Does this create a useful output?), efficiency (Would having these outputs have saved time?), and potential for bias (What unintended consequences might arise from these outputs?) [6]. Evaluators were also asked to give an overall score on the quality of the ChatGPT output (Overall, how good would you say this output is?). Post review, a group debrief was conducted, using a semistructured interview guide to facilitate discussion regarding perceptions of outputs and rationale for ratings.

### Ethical Considerations

Ethical considerations helped guide the initial development of research methods and reduce potential risks for participants in the original study implementation with the PAMS intervention [7]. Recreating the technical development of a system previously built as part of the dDPP randomized controlled trial (NCT04049500) has not introduced any new risks to patients. Patients were not involved in this research examining the use of GenAI in the development of digital health care solutions. No patient data was used in the prompt generation phase.

Regarding ethical considerations for the methods used in this paper, as an attempt to mitigate evaluator biases, we worked with a diverse team of evaluators who were aware of the initial study but were not necessarily involved in the technical development. Additionally, we understand the limitations and concerns of the use of ChatGPT including possible hallucinations and incorrect answers. Thus, we emphasize the need for human expertise to identify correct and incorrect outputs and have flagged this as a study consideration. When developing the GenAI-based solution, we used the same considerations for data security, patient usability, accessibility, and data privacy used in the original human-developed solution.

### Prompt Generation Framework

**Figure 1:**
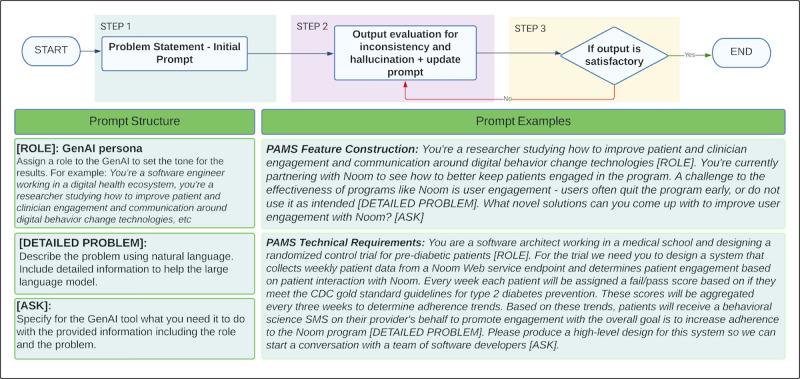
ChatGPT prompt structure and prompt examples. CDC: Centers for Disease Control and Prevention; GenAI: generative artificial intelligence; PAMS: personalized automatic messaging system.

Prompt engineering focuses on the skill of designing and creating effective prompts that guide ChatGPT to produce the best possible output for your task. We followed existing literature [8-11] combined with our expertise and experimentation to provide a framework that yields the best result when developing a digital solution like PAMS (Figure 1).

## Results

### PAMS Concept and User Experience Generation

#### Overview

Core components of the PAMS intervention were conceptualized and designed via an underlying behavior change theory, design principles and personas, and a message content library.

#### Underpinning Behavior Change Theory and Approach

##### Human-Generated Solution

**Figure 2:**
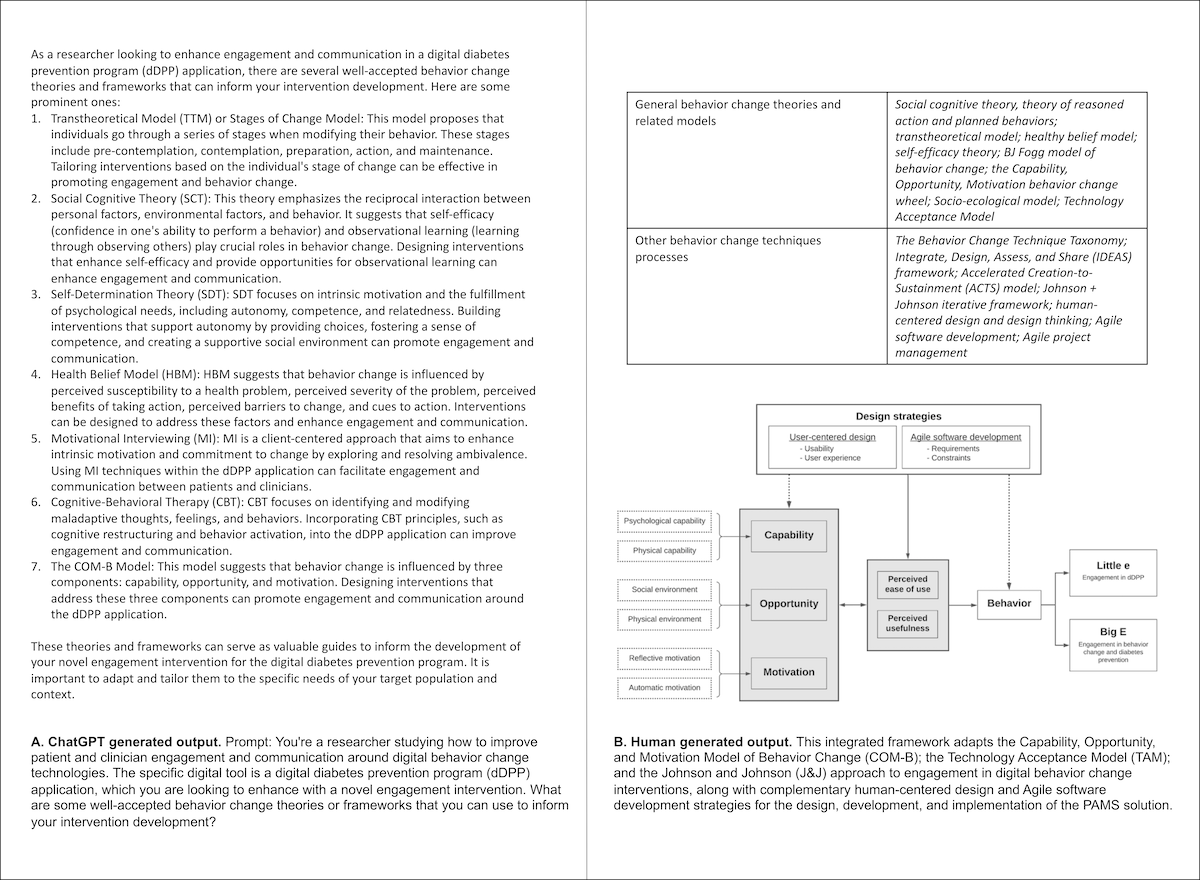
Underpinning behavior change theory and approach outcome of ChatGPT vs human-generated output. ACTS: Accelerated Creation to Sustainment; BJ: Brian Jeffrey; CBT: cognitive behavioral therapy; COM-B: capability, opportunity, and motivation model of behavior change; dDPP: digital diabetes prevention program; HBM: health belief model; IDEAS: Integrate, Design, Assess, and Share; J&J: Johnson and Johnson; MI: motivational interviewing; SCT: social cognitive theory; SDT: self-determination theory; TTM: transtheoretical model.

Leveraging behavior change literature review and interviews with behavior change theory content experts (n=4), the research team initially identified ten unique behavior change theories and six process models that were considered to be an appropriate fit for the aims of the overall intervention. A unique model was developed that captured (1) the relevant underlying behavior change theory, (2) implementation strategies, and (3) unique contexts of the technology environment (Figure 2A).

##### GenAI Solution

 When prompted, ChatGPT identified seven relevant well-accepted behavior change theories and frameworks to inform a “dDPP support intervention” (Figure 2B). It did not provide information on the transtheoretical domains framework, or the taxonomy of behavior change techniques, but when prompted on these, identified both as acceptable strategies for use.

#### User Experience: Design Principles, Personas, and Messaging Content

##### Human-Generated Solution

**Figure 3:**
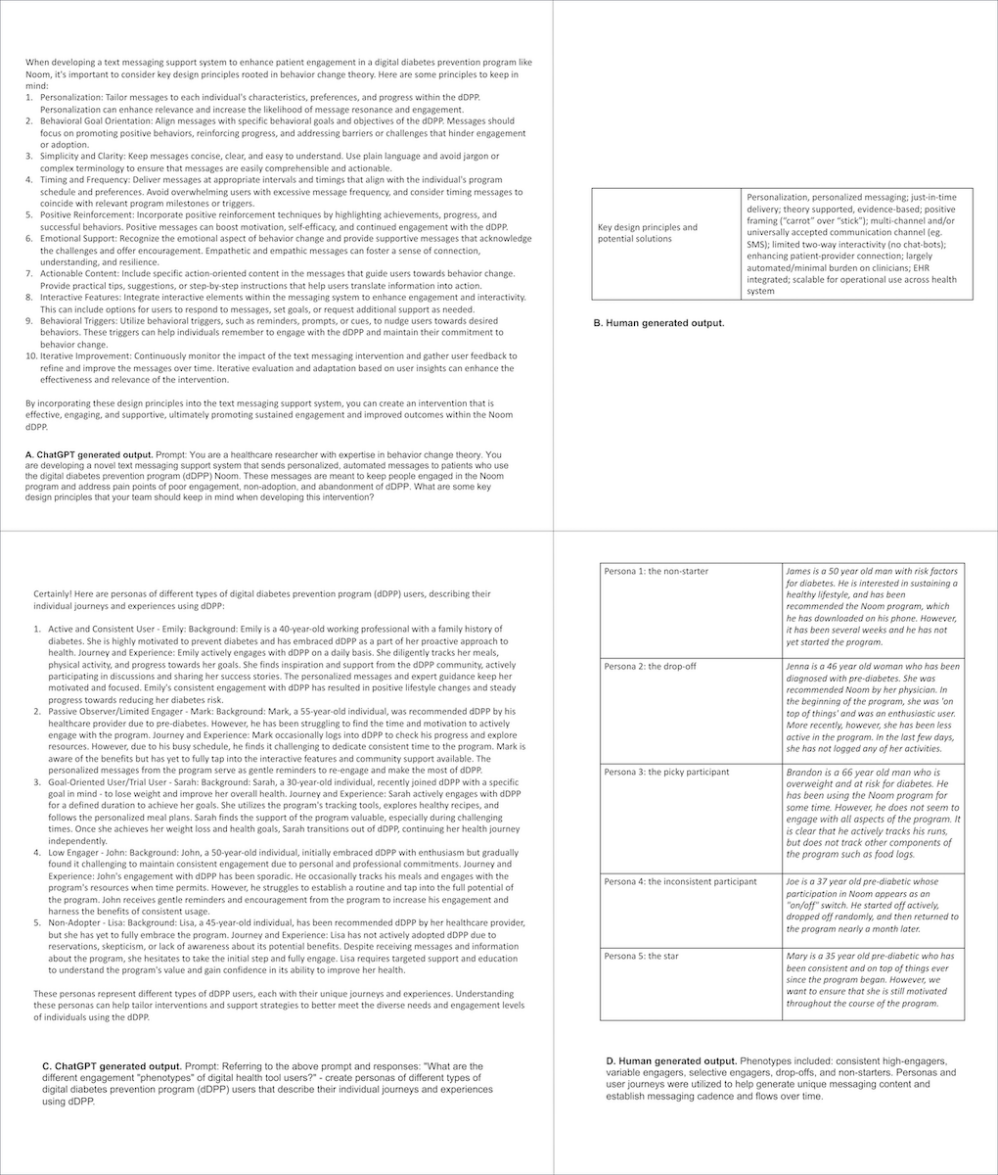
User experience: design principles, personas, and messaging content outcome of ChatGPT vs human-generated output. dDPP: digital diabetes prevention program; EHR: electronic health record.

The research team used a human-centered design approach to identify key design principles, defined as the set of considerations that form the basis of the PAMS product (Figure 3B). These were developed from insights gathered via a review of relevant digital behavior change research, consultation with content and theoretical experts in digital health and implementation science (n=3), and two group interviews (n=9). From these insights, five relevant fictional personas were designed to capture the various phenotypes of user engagement with the commercial dDPP, along with unique user journeys developed to describe their projected engagement with the program over time (Figure 3D). Overall, over 193 unique messages were developed, each grounded by a relevant behavior change technique and tailored to an individual phenotype’s user journey. These elements were continuously revisited and refined during the testing phases of the dDPP research. This included a 6-month near-live user testing phase consisting of nine patients engaging with various iterations of the PAMS prototype, and a 12-month live single-arm pilot phase consisting of 25 patients using PAMS-beta with the commercial dDPP platform.

##### GenAI Solution

ChatGPT was prompted from multiple perspectives (researcher, clinician, and patient) to identify key design principles (Figure 3A) and sample solutions for the PAMS intervention. It also provided common engagement phenotypes for digital health tool users, based on patterns of use, frequency, duration, and “other elements.” Of note, nonadopters were not identified within the initial round of phenotypes. ChatGPT also developed personas for each of the identified engagement phenotypes, including persona names, backgrounds, and individual journeys. ChatGPT was able to produce five to ten unique messages targeted toward each phenotype and to adapt these messages based on various additional prompts. The user types or personas generated by ChatGPT are consistent with the human-generated users and cover all the phenotypes identified in our previous research (eg, mapping to a specific behavior change technique and reflecting a key design principle; Figure 3C).

### PAMS Technical Development

#### Overview

The technical development includes a PAMS requirements document and architectural design and code.

#### Technical Requirements (User Stories)

##### Human-Generated Solution

**Figure 4:**
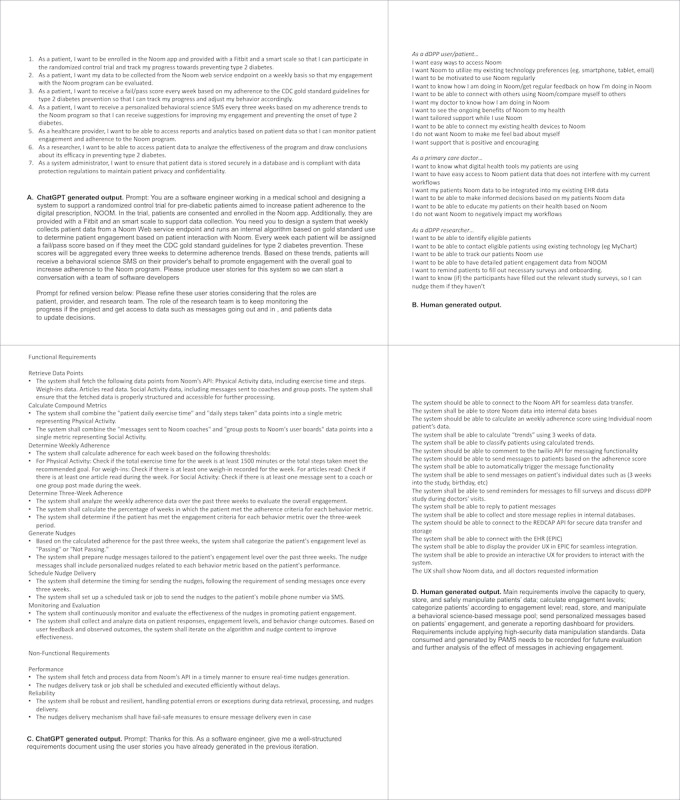
User stories and technical requirements outcome of ChatGPT vs human-generated outputs. API: application programming interface; CDC: Centers for Disease Control and Prevention; EHR: electronic health record; PAMS: personalized automatic messaging system; REDCap: Research Electronic Data Capture; UX: user interface.

Following the data collection and intervention design period, we created, as a team, a series of user stories (Figure 4B) which were followed by system requirements to describe the intended use cases, features, and challenges of the proposed PAMS software. Initial system requirements represent the “minimum viable product” that was developed, piloted, and further refined (Figure 4D). Our development team followed software engineering principles to generate the requirements document.

##### GenAI Solution

We used the output of the “feature construction phase” to inform the GenAI output for requirements. During the initial stages of the prompting phase, we refrained from suggesting solutions, allowing ChatGPT to generate potential solutions autonomously. We reviewed and evaluated these outputs, eliminating impractical or incompatible solution paths that did not align with the intentions or capabilities of our team. Once we reached a satisfactory outcome but faced uncertainty regarding the next steps, we instructed ChatGPT to assume a different “personality” (eg, software architect) and used the previous outputs as a foundation for the new role’s initial prompts. Throughout this process, we encouraged each “personality” to seek clarifications by asking questions and provided feedback without biasing toward any predetermined solution. We repeated this process at least four times for each personality type, engaging in a back-and-forth roleplay with multiple personalities (researcher, architect, and developer), transitioning to a different personality when it became evident that the current one could no longer progress without additional feedback (Figures 4A and 4C).

#### Architectural Design

##### Human-Generated Solution

**Figure 5:**
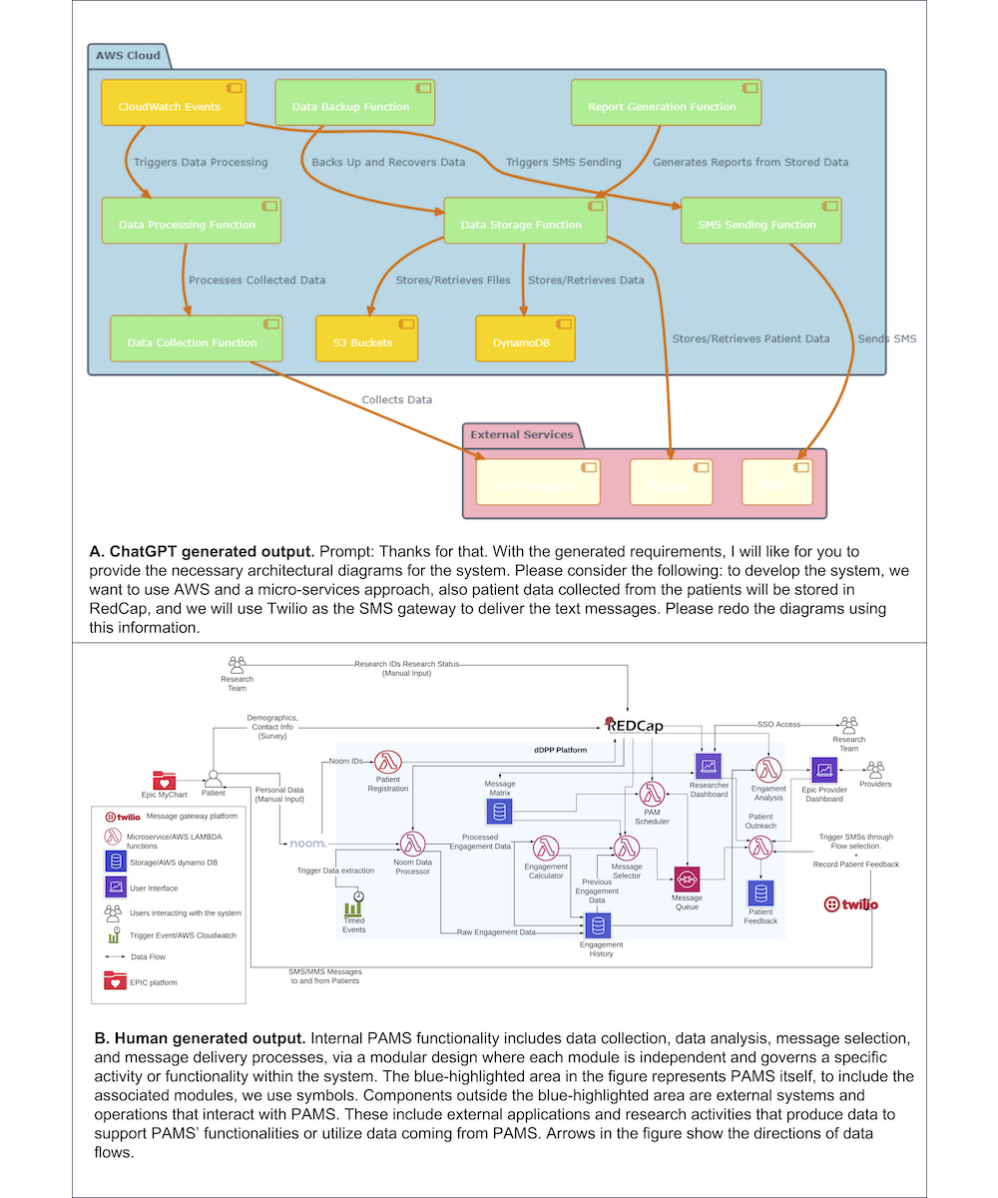
Architectural design for PAMS. ChatGPT vs human-generated output. AWS: Amazon Web Services; dDPP: digital diabetes prevention program; PAMS: personalized automatic messaging system; REDCap: Research Electronic Data Capture.

After the requirement phase, our software development team developed the PAMS architectural diagram, which is a graphical representation of the system that includes (1) a set of components (eg, a database and computational modules) that will perform a function required by the system; (2) the set of connectors that will help in coordination, communication, and cooperation between the components; and (3) conditions for how components can be integrated to form the system (Figure 5B).

##### GenAI Solution

For the GenAI-generated architectural design, we leveraged the outputs of the requirement phase and the available ChatGPT plugins to designate the GenAI model as a software engineer and proceeded to develop an architectural diagram. During this process, we engaged in iterative prompting and provided explicit instructions to ChatGPT, specifying the use of Amazon Web Services (AWS) for development, integration of external systems such as Twilio (Twilio Inc) and REDCap (Research Electronic Data Capture; Vanderbilt University), and the adoption of a microservice approach to facilitate the efforts of our development team (Figure 5A).

#### Code

##### Human-Generated Solution

**Figure 6:**
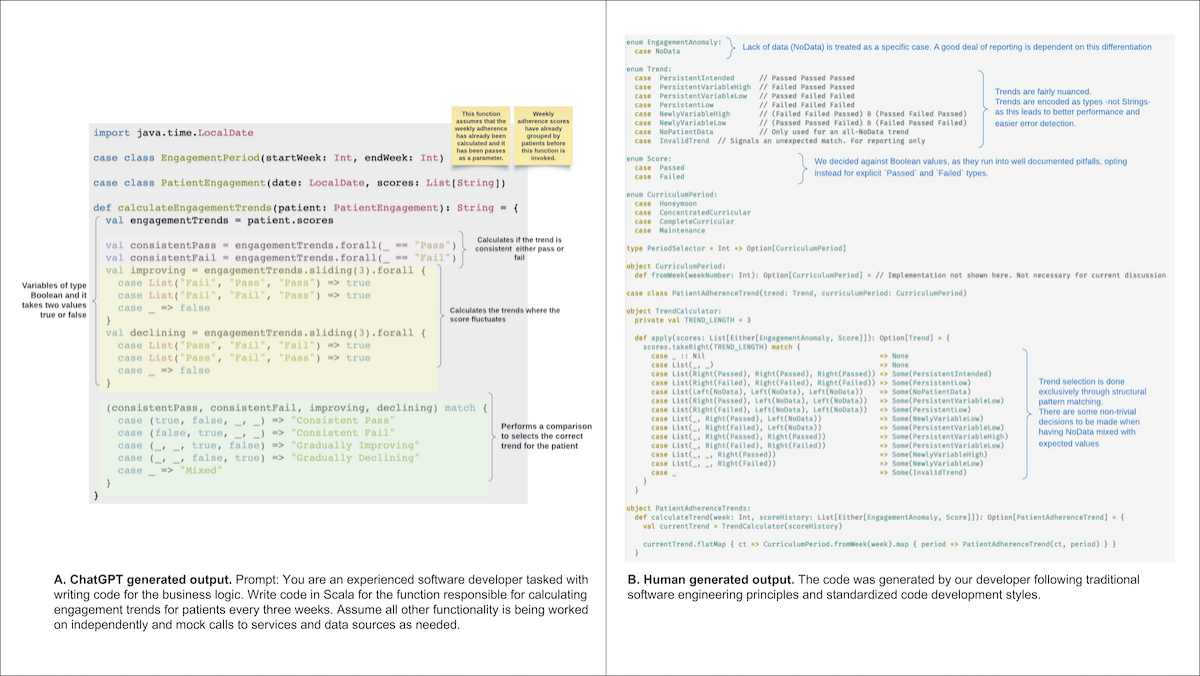
Code for the function that calculates patient adherence and engagement trends. ChatGPT vs human-generated outputs.

PAMS components include several lambda functions that execute its engagement or adherence algorithm, messaging, and data manipulation functionalities. Most of the functions are coded and developed using Python (Python Software Foundation) and Scala (École Polytechnique Fédérale Lausanne) as programming languages. AWS was used for the development of PAMS [12]. Our developers followed our microservice approach design using an event-driven model [13,14]. The main components of PAMS are AWS lambda functions which are triggered by different events such as updates to S3 buckets, modifications on DynamoDB (AWS) tables, or CloudWatch (AWS) events. External interactions of PAMS use application programming interface calls, which secure effective data transfer (Figure 6B).

##### GenAI Solution

To facilitate the generation of the coded solution using ChatGPT, we assigned the role of a software engineer to the model and specifically requested it to generate Scala code for a specific functionality, namely the “calculate engagement trends” function. Consistent with the iterative nature of the GenAI-based software development process, we engaged in a back-and-forth interaction with ChatGPT, iterating over the prompt and its output while providing expert guidance to ensure optimal results. While allowing ChatGPT to generate free text, we evaluated each output for accuracy and adherence to the desired specifications (Figure 6A).

### Internal Review of Human Vs GenAI Outputs

The 11 evaluators participated in the output review process. All had familiarity or prior exposure to the original PAMS intervention. Overall, evaluators rated the ChatGPT-produced outputs as positive for the theoretical background and design phase in terms of understandability, usability, novelty, relevance, and efficiency. For these two components, the question about completeness showed the most variability with divided opinion among “agree” and “disagree” and the bias was mostly categorized as “neither agree nor disagree.” For the first part of the technical development (user stories and requirement documents), most of the raters found the ChatGPT output positive in terms of understandability, usability, and relevance. In terms of completeness and novelty, requirements were better rated than the user stories which represent an interesting output since requirements are derived from the user stories. We hypothesize that our raters were expecting better user stories, but once these were defined, they considered ChatGPT to be effective at turning these into the requirements. In terms of bias, similar to the theoretical background and design phase, the most popular answer was “neither agree nor disagree.” For the more technical pieces of the development that required software engineering knowledge, specifically the architectural diagram and code elements, results showed the highest N/A responses. These higher levels of N/As were associated with lower levels of expertise (eg, coding experience) since only 2 of the 11 evaluators had computer science backgrounds. However, the overall score excluding the N/As was positive for the technical component.

## Discussion

### Results Summary

This study leveraged ChatGPT-4 to recreate content features and software development of PAMS. ChatGPT served as a usable facilitator for researchers engaging in the software development life cycle, from product conceptualization to feature identification, and user story development to code generation. GenAI technologies facilitated effective communication and understanding within our multidisciplinary team by providing well-described features and supporting the role of a software engineer. Our findings indicate that the ChatGPT-generated output is comprehensive, albeit with occasional ambiguities that required clarification or adjustment by the research team. The ChatGPT-generated output exhibited a high level of accuracy in capturing the intended requirements. We found that ChatGPT supported a highly efficient development process, producing over 5 days what initially required more than 200 human hours from content and technical experts. The results suggested that by efficiently prompting ChatGPT and leveraging the expertise of our team, we could have significantly reduced the time we invested in initial system modeling and conceptualization phases as well as technical phases of software development (coding). Overall, GenAI technologies like ChatGPT offer a promising approach to efficient software development.

While promising, some significant limitations to ChatGPT’s outputs should be noted. In the design phase, while ChatGPT was able to provide general guidance in tool design (eg, app vs web-based vs EHR solution) it was unable to provide evidence to support its rationale for these choices. This lack of reference support has been well-documented and has a material impact on researchers looking to build upon an evidence base for their health technology interventions. Similarly, when asked to provide theoretical frameworks to support behavior change, it offered only a partial list, initially excluding the COM-B (capability, opportunity, motivation, behavior) model upon which the original PAMS intervention was based, and needed prompting from our behavior change expert to provide more specific guidance. In the context of code generation, we focused on testing a specific function, namely the Calculating Patient Engagement feature, which is the core functionality of our software. Initially, we tasked ChatGPT with generating a function to compute a 3-week patient engagement trend. However, the initially generated code deviated from the intended objective and instead calculated a weekly engagement score. Through subsequent iterations, we were able to obtain the desired code. However, the initial attempts exhibited nonidiomatic constructs and contained bugs (no efficient loops and wrong logic). Finally, we observed that ChatGPT overlooked certain suggested features during the design phase, resulting in the generated code occasionally demonstrating unnecessary complexity and disregarding some of the best practices and features of the target programming language. We believe that further iterations would have improved the code quality, encompassing better adherence to coding standards and the inclusion of desired business features, such as handling edge cases and capturing more nuanced engagement trends. Nevertheless, we reached a point of diminishing returns with ChatGPT where we determined that engaging an experienced developer would have expedited the code generation process and ensured a more robust implementation.

These limitations highlight the ongoing importance of human expertise in the development process, especially in scenarios where theoretical expertise, intricate coding practices, and business-specific requirements are involved. The lack of rationale to support the generated results shows the value of having human experts on the team who can interpret the results. ChatGPT needs to be used as a support tool but not the source of truth; thus, we always trusted and relied on human experts to validate the ChatGPT-generated results before moving to the next phase. Overall, it is important to have human experts in the system development process to guide the outputs in terms of reprompting the system (support the decision-making on acceptable output) and ensuring their accuracy. Moreover, results are highly dependent on the quality of the prompts which emphasizes the role of prompt engineering. The results show that well-structured prompts (role + problem description + ask) that infuse human expertise into every iteration are key to obtaining good results (Figure 1). As part of our prompt framework described in the methodology section, results showed that detailed problem explanations, clear asks, and roleplaying are an excellent combination to guide accurate results. We suggest asking ChatGPT questions using different roles, asking for clarification if needed, and in cases of wrong outputs, redirecting the prompts.

### Related Work

There is near-universal interest in understanding the impacts of GenAI and large language models (LLMs) on human social structures, including the experience of work and the production of work-related outputs in health care and more broadly [15,16]. In health care, LLMs are poised to impact everything from care delivery experience, diagnostic reasoning and cognitive skills, training and education, and the overall composition of the workforce [17]. These theoretical disruptions are tempered, however, by acknowledging that in its current state, GenAI tools remain suboptimal, with ongoing issues in accuracy, reliability, usability, cost, equity, and ethics.

In commercial spaces, ChatGPT-enabled products designed to assist with coding and software development are already being developed (eg, OpenAI Codex [OpenAI] and CodeGPT [CodeGPT]). These tools can help generate novel code, debug and analyze code issues, assist in code refactoring, and provide code documentation. As yet, however, their usefulness in terms of quality has not been extensively evaluated, and costs and other considerations may make them inaccessible to health care researchers. ChatGPT-enabled tools for front-end design (eg, integrating ChatGPT with Figma [Figma, Inc]), user testing (including synthetic user testing), and prototyping have also been created, all allowing health technology research teams with limited design resources to take advantage of tools from product and experience design to create their interventions. Overall, commercial LLMs have been demonstrated to improve worker efficiency and productivity, through “co-pilot” support services that automate low-skills tasks, organize and present information, and surface insights [18]. Brynjolfsson et al [18] found that a ChatGPT-supported tool providing conversational guidance for customer support agents increased worker productivity by almost 14%. The authors further found that these productivity benefits accrued disproportionately to less-experienced and lower-skill workers, allowing less-skilled or newer workers to experience more rapid gains; the authors posit that high-skill workers may have less to gain from artificial intelligence assistance due to tacit knowledge reinforcement rather than new knowledge or skill development. Our work suggests that both less-experienced, lower-skill workers and high-skill workers can benefit, with novices benefitting more from new knowledge (if accurate) and skill development and experts benefiting from knowledge validation and offloading of high-effort low-value tasks.

In the academic computer science literature, ChatGPT has been evaluated as a tool for collaborative software design [4], including to improve code quality refactoring, requirements elicitation, and general design solutions [5], and fix programming bugs [19]. Similar findings are reflected in our work, including the caveats of requiring human oversight. Other authors have identified important ethical issues in using GenAI solutions for software engineering, which were not considered in this study [20].

Within health care, a growing body of research has explored the feasibility of GenAI tools (mostly ChatGPT) in a variety of use cases, including answering patient questions [3,21], creating suggestions to optimize clinical decision support [22], generating a history of present illness summaries [23], and overall examination performance [24]. In general, these papers find promising signals for the accurate and acceptable use of GenAI tools, but with many current-state caveats for their optimal, safe, and scaled use. Key areas of concern include reliability (particularly around hallucinations and citation fabrication), reproducibility, and recency of data inputs. While research in this area will continue to grow, as more test cases comparing GenAI performance to that of clinical staff will be undertaken, further work is needed to create validated and generalizable outcome measures. Future work must also ensure that the variety of GenAI tools (including general commercial LLMs, health care–specific LLMs, and internally developed tools) are equally evaluated.

### Limitations

There are several limitations to this study. First, no research team members have expertise in prompt generation for GenAI tools; as a result, our prompting reflects the a priori perspectives, biases, and knowledge gaps of our team, and are therefore particularly subject to issues of framing, recall, and confirmation bias that may influence the interpretation of the results. Second, our research team members, who acted as prompt engineers in this study, were highly familiar with the project and participated in the human-based design process; thus, they were aware of what deviations from human-based design to address by reprompting the system. As a result, we have introduced bias in the prompting process and results reflect higher accuracy. Third, the absence of robust tools to objectively measure the “quality” of current ChatGPT outputs poses challenges to accurately and objectively assess its performance. Furthermore, in this case, the output reviewers were not blinded to the human vs ChatGPT outputs, given the complexity of this study and the difficulty in providing enough research context to support independent blind review. Finally, broader limitations of the technology, such as potential hallucinations and concerns about behavioral changes of responses over time, deserve acknowledgment, as they could have implications for the practical applications and long-term viability of GenAI in health care research contexts. Future research efforts should address these limitations to enhance and replicate our findings.

### Implications and Future Directions for Exploration

We are considering several future directions for the use of ChatGPT in our digital health intervention development. We envision increasing our expertise in prompt engineering (add expert prompt engineers to the team) to actively use ChatGPT to further develop PAMS features, particularly for additional messaging content. We anticipate this will save our research team considerable time and effort. We may also use ChatGPT to facilitate more time-consuming aspects of our research documentation, including both coding documentation and larger research archival work (eg, meeting minutes and recording intervention decision-making). Overall, we feel ChatGPT and related tools can be effectively leveraged within health care technology research teams with a spectrum of technical expertise, serving to both augment existing skills and supplement skill gaps. For those with expertise in computer science or programming, we imagine ChatGPT can assist by automating high-effort, low-impact tasks or repetitive work that is considered important but often deprioritized as more urgent tasks arise (eg, code documentation). For those without preexisting programming skills, we imagine ChatGPT can offer technical support, including educational tools and skill-building opportunities. Overall, this process will both validate existing knowledge and create new knowledge for teams, as well as potentially improve interteam communication and collaboration.

### Conclusions

In this study, we explored the use of the GenAI tool ChatGPT to recreate a novel digital behavior change intervention which our research team had previously developed to support patient engagement and adherence to a commercial dDPP. Specifically, we reviewed and evaluated the capacity and limitations of ChatGPT to support digital health research intervention ideation, design, and software development, finding it a feasible and potential time- and resource-saving tool to support research teams in developing novel digital health products and technologies. At the same time, we identified gaps in ChatGPT outputs that may limit its effective use for both novel and advanced technology developers, particularly around the completeness of outputs. Future directions will include the development of more targeted artificial intelligence–based tools to support health care researchers with all levels of software or engineering skills, as well as the development of improved tools to objectively evaluate GenAI outputs.

## Abbreviations

AWS: Amazon Web Services
COM-B: capability, opportunity, motivation, behavior
dDPP: digital diabetes prevention program
GenAI: generative artificial intelligence
LLM: large language model
N/A: not applicable
PAMS: personalized automatic messaging system
REDCap: Research Electronic Data Capture
